# Quantitative evaluation of recruitment strategies in a cluster-randomized trial: segmented regression and cost analysis from the AOK-Family + study

**DOI:** 10.1186/s12874-025-02734-8

**Published:** 2025-11-28

**Authors:** Nicola Litke, Michel Wensing, Lina Hermeling, Manuela Bombana

**Affiliations:** 1 Department of General Practice and Health Services Research, University Hospital Heidelberg, Im Neuenheimer Feld 130.3, Heidelberg, 69120 Germany; 2 Department for Prevention , AOK Baden-Württemberg, Presselstraße 19, Stuttgart, 70191 Germany

**Keywords:** Lifestyle-related risk factors, Pregnancy, Behavior change intervention, Recruitment, Linear segmented regression analysis

## Abstract

**Background:**

Recruitment of participants for preventive health intervention studies remains a significant challenge: Approximately 19% of studies are discontinued due to insufficient participant numbers, and one in three extends its recruitment period. This study aimed to examine the impact of recruiting measures on the recruitment rate over time and to describe costs of these strategies to inform future study planning and optimize resource allocation.

**Methods:**

A cluster-randomized controlled trial (“AOK-Family+” study) was conducted from April 2023 to June 2024 in southwestern Germany. The intervention focused on reducing lifestyle-related risk factors (LRRFs) among pregnant women and women planning a pregnancy. Analog recruitment strategies included e.g., printed magazines, flyers and digital recruitment strategies included e.g. social media ads, influencer marketing. For 255 interested women, contact details, recruitment source, and participation status were documented and analyzed. A segmented linear regression analysis was used to identify turning points in application trends. Direct costs were calculated based on internal project budget tracking.

**Results:**

The targeted sample size was not reached despite substantial investment in recruitment measures. The highest number of applications resulted from analog strategies—especially printed AOK magazines (35.5%)—followed by influencer marketing (23.6%). The segmented linear regression analysis identified three significant increases in application rates, the first coinciding with the magazine distribution and the second with the influencer marketing on Instagram. Social media marketing showed a short-lived effect, with application rates dropping immediately after posts ended. Total costs amounted to 99.334 € equaling 389,54€ per application and 534,05€ per actually enrolled participant.

**Conclusion:**

Health magazines proved to be the most cost-efficient and sustainable recruitment strategy. Influencer marketing led to high reach and initial spikes in engagement but had limited long-term impact. While digital measures generated many clicks, only a small fraction translated into study participation—indicating a pronounced intention–behavior gap and procedural barriers in the enrolment process. For future studies, a mixed-methods recruitment strategy is recommended combining wide digital outreach with personalized, trust-based communication, ideally through healthcare professionals such as gynecologists and midwives, to reach women in early pregnancy and reduce participation barriers.

**Trial registration:**

The German Clinical Trials Register DRKS00027804. Registered on 2022/01/12.

## Background

The recruitment of study participants in human research on preventive interventions is described as challenging, as 19% of studies had to be terminated due to recruitment issues and one-third of studies extend the recruitment period [1, 2]. Pregnant women — including those in early pregnancy — are often considered a hard-to-reach group due to time constraints, competing priorities, and privacy concerns, and recruitment rates vary considerably between different pregnancy-related intervention studies [3–6]. Recruitment strategies vary widely in effectiveness depending on the study context and the characteristics of the target population. Personal approaches, such as direct contact through health professionals, phone calls, or face-to-face conversations, are frequently associated with higher response rates and stronger trust-building, particularly in groups with lower health literacy or heightened skepticism toward research participation [7, 8]. However, personal contact is resource-intensive, limited in scalability, and often constrained by the availability and willingness of health professionals to act as recruiters, which can hinder its feasibility in large-scale public health interventions [8, 9]. At the same time, digital strategies such as social media advertising, online panels, and targeted web-based campaigns have been shown to reach broader and younger audiences at lower cost, yet their effectiveness varies considerably across settings, with some studies reporting high initial engagement but low conversion rates into actual enrolment [10, 11]. Moreover, digital strategies and social media recruitment is described as a method to recruit study participants and hard-to-reach target groups, including women exposed to lifestyle-related risk factors (LRRFs), e.g. alcohol or tobacco abuse [12], and has also shown promises in mental health research [13] and in pregnant women [14]. Within these literature reviews, cost-effectiveness of social media recruitment varies depending on the respective study setting, target population, and communication channels employed.

LRRFs can affect maternal health and pregnancy outcomes. Adverse health behaviors during pregnancy - such as smoking or alcohol consumption - can have profound irreversible effects, potentially causing lasting harm or even mortality for the unborn child [15, 16]. Furthermore, health literacy is a key determinant influencing risk behaviors during pregnancy (Morrison et al., 2019). Promoting healthy behavior during pregnancy is crucial to reduce health-related risks for the embryo, foetus, and expectant mother, and to minimize long-term maternal and child morbidity and mortality [17–20]. Specifically, diet and physical activity can reduce gestational weight gain, risk of gestational diabetes, risk for preterm delivery, and overall adverse maternal outcomes [21, 22]. However, LRRFs are socially patterned: women with a lower socio-economic status (SES) are at higher risk of unhealthy behaviors during pregnancy [23–26] and experience worse health outcomes [27, 28]. At the same time, they participate less often in preventive interventions, which may further reinforce existing health inequalities. Thus, recruitment strategies in pregnancy-related intervention studies are not only critical for achieving sufficient sample sizes, but also for ensuring equitable access to prevention programs. Understanding which recruitment strategies are effective and cost-efficient is therefore directly relevant to promoting health equity in maternal and child health. In addition, recent evidence recommends involving multipliers such as general practitioners and midwives [7] as well as community-driven approaches for hard-to-reach groups [29]. In this study, recruitment was based on established practices of AOK Baden-Württemberg. The subsequent section outlines how these established practices were operationalized within the recruitment strategy and implemented throughout the study.

The project “AOK-Family Happiness Plus for Individual Prevention and Strengthening of Resources in Pregnancy and Optimization of Education in Gynaecological Care” (AOK-Family +) includes a consultation addressing LRRFs during pregnancy for pregnant women or women in preconception period [30]. To reach this target group, a broad spectrum of recruitment strategies were included such as social media content and printed media.

The aims of this article were (1) to examine the impact of recruitment strategies on the recruitment rate over time and (2) to describe costs of these strategies to inform future study planning and optimize resource allocation.

## Methods

The project “AOK-Family +” includes a cluster-randomized controlled trial addressing LRRFs through preventive health consultations for pregnant women and women in the family planning phase in southwestern Germany. The study is conducted by the health insurance company AOK Baden-Wurttemberg (AOK BW) and the Department of General Practice and Health Services Research of the University Hospital Heidelberg which is conducting the accompanying outcome evaluation and process evaluation [30]. This process evaluation included *N* = 12 semi-structured interviews and 1 focus group, using a semi-structured interview guide. For conducting the intervention study, pregnant women and women in the family planning phase were recruited from April 2023 to June 2024 via various different approaches.

### Study setting

The German health insurance company AOK BW and the Department of General Practice and Health Services Research of the University Hospital Heidelberg developed a consulting intervention for pregnant women and women in family planning phase. The consultation focused on LRRFs during pregnancy within the areas of nutrition, physical exercise, stress management and the consumption of alcohol, tobacco and other drugs. The intervention consisted of a one-hour, one-on-one consultation that could be conducted either in person (study arm 1) or via an online meeting (study arm 2). Participants received questionnaires directly before (t0) and after (t1) the consultation. A third study arm consisted of a waiting control group that received the same baseline questionnaire but no consultation and no t1 questionnaire. Furthermore, all participants (all study arms) received a six-week follow-up questionnaire (t2). Participants of the waiting control group were offered a consultation of their choice (analogue or digital) after completing the follow-up questionnaire.

The intervention was conducted from April 2023 to July 2024 in local health centres of AOK BW within the German south-western federal state of Baden-Wurttembergs. The 14 district directorates of AOK Baden-Wuerttemberg in Southwest Germany were randomized to one of three study arms: on-site intervention, online intervention, or control. Randomization was computer-generated using a simple cluster-randomized design (randomizr package in R, version 1.3.5) and performed by an independent operator prior to enrolment. Allocation was concealed from the study team. Counsellors were assigned according to their place of employment, and insured women were linked to the intervention arm of their residential district directorate. Thus, district directorates constitute the randomized units. Primary outcomes of the evaluation were pregnancy-related health knowledge and health behavior. The accompanying process evaluation aimed to identify factors influencing further implementation of the intervention and included interviews and a focus group with consultants. More details about intervention components and the study conduction are reported in the study protocol [30].

### Study population

The planned sample size for the study was *n* = 638 participants. The target group included pregnant women and women in childbearing age who were between 18 and 49 years and able to give consent. Only women insured by AOK BW were eligible. Furthermore, participants were required to have sufficient proficiency in German to speak and read the language adequately. All eligible individuals who were interested in the study were recruited for the intervention study.

### Recruitment strategies

The recruitment strategies included a variety of strategies to reach the target group. The recruitment strategies were grouped into two main categories: (1) analogue measures, including printed magazines, flyers, and personal approaches in AOK customer centers and health centers, and (2) digital measures, including social media campaigns, influencer marketing, and advertisements on websites, apps, and newsletters. These sets of measures were implemented at different time points before, during, and towards the end of the intervention, as illustrated in Fig. 2.

Recruitment was conducted by the AOK BW team who planned, funded and deployed all recruitment strategies The AOK BW team comprised of eight staff members (prevention and communication specialists). Most strategies were part of AOK BW’s regular advertising, while selected measures (e.g., influencer marketing, targeted social media ads) were adapted specifically for this study. Some strategies can be terminated exactly, but with most strategies, a time period has to be stated. For example, health magazines were sent to AOK BW clients at the beginning of the respective calendar quarter with a variable mailing process. For these strategies, an approximated time period has been estimated.

For study participation, interested women were asked to call a service hotline of AOK BW for which the contact information could be found on the AOK BW homepage. If the call was outside of the opening hours of the hotline, interested women were asked to leave their contact information on an answering machine and the study team called them back as soon as possible. Within this first phone call, criteria for study eligibility were verified, participants were informed about study procedures and, after their consent, participants were assigned to a study arm via cluster randomization.

The unit of randomization was the 14 district directorates of AOK Baden-Wuerttemberg, which were randomly allocated to one of three study arms (on-site counselling, online counselling, or waiting-list control). Individual participants were automatically assigned to the intervention arm of their residential district directorate.

Eligible participants were women aged 18–49 years who were either pregnant or in the family-planning phase, insured with AOK Baden-Württemberg, able to provide informed consent, and sufficiently proficient in German to complete study materials. Women outside the age range, not insured with AOK Baden-Wuerttemberg, or unable to provide consent or understand German were excluded.

Furthermore, these callers were asked for the respective recruitment strategies they based their decision to apply for study enrolment. In general, it cannot be excluded that a person was exposed to several of the recruitment strategies s and some participants reported more than one recruitment strategy. The strategy used for this analysis was the one that formed the basis for the decision to participate in the study.

Following the first phone call, a respective consultant was informed by the study team and contacted the participants for their consultation if they were in study arm 1 or 2. Also, participants received the t0 baseline survey-link via email during that time period.

### Data sources and measures

Data sources comprised (i) routine records from AOK Baden-Württemberg documenting the timing, scope, and content of recruitment measures, (ii) project management files detailing implementation processes, and (iii) administrative datasets on both expenditure and participant enrollment.

Two social media advertising campaigns (“flights”) were managed by an external media agency commissioned by AOK BW. For these, campaign analytics were summarised descriptively, including story views, link clicks, website visits, and applications.

All financial data were consolidated in a dedicated subsection. Costs were calculated in euros (€) and systematically classified into predefined categories: personnel, media production, print materials, distribution, and digital advertising (e.g., paid social media campaigns). All costs for the intervention provider directly linked to recruitment activities were obtained from AOK BW’s accounting records. Costs are reported in nominal terms for the years 2023 and 2024 (no indexing applied) and covered the period from April 2023 to July 2024 and were sourced directly from unmodified internal account records and invoices from external agencies. Costs were aggregated according to the recruitment measures listed in 2. To ensure transparency, expenditures attributable to AOK BW’s routine marketing activities were distinguished from study-specific adaptations. Both absolute and relative cost distributions are reported to enable a comprehensive assessment of resource allocation.

No questionnaires, interviews, or surveys were used for the data reported here. While self-developed questionnaires were used in the AOK-Family + study to assess health outcomes, these results will be reported in separate publications.

### Data-analysis

This article reports a prospective observational analysis of recruitment dynamics, examining how recruitment rates developed over time in relation to the applied strategies. In addition, the direct costs of each recruitment approach are presented to inform future study planning and resource allocation.

Recruitment outcomes were reported descriptively. To visualize the timely aspects of recruiting strategies, the date of the first phone call was used. The time period for recruitment is measured in days. For data analysis, day 1 is defined as the day of the first phone call (24.04.2023). As officially, the trial started at the beginning of April 2023, the time period before the first recruitment is summarized as “day 0”. This period of time is not included in data analysis but is used to describe and terminate recruitment measures that started parallel to the intervention start. For these purposes, data was analysed in Microsoft Excel Version 1208 (Microsoft Office LTSC Professional Plus 2021).

Analyses were conducted using R 4.5.0 [31]) with the segmented package [32].Segmented linear regression was applied to detect potential breakpoints in the time series of recruitment inflow and to estimate changes in slope across segments. The principle of the method lies in estimating regression lines within intervals separated by breakpoints, with the difference-in-slope parameter quantifying changes in the trajectory between segments [32].

Analysis of residuals was undertaken to test model assumptions. A Q-Q plot of residuals suggested a reasonably normal distribution. A plot of predicted values versus residuals looked largely reasonable but suggested potential heteroscedasticity in some segments. Multicollinearity analysis (variance inflation factors under R package car) showed that the breakpoints were reasonably independent. Models` parsimony was displayed using a scree plot of the Akaike Information Criterion (AIC) values, where lower values indicated better model fit while avoiding unnecessary complexity.

For each estimated breakpoint, 95% confidence intervals were calculated to assess location uncertainty. The Davies test for non-constant regression parameters was conducted separately for each model, in order to compare the increase in the number of breakpoints with the previous model. Davies’ test showed significant values for models up to 5 breakpoints, but not for the model with 6 breakpoints. Models were estimated using the default maximum likelihood algorithm implemented in the segmented package.The model with the best goodness of fit the was used, considering models with up to six breakpoints. This number was not pre-specified as a theoretical upper bound but reflects the practical limit of model fitting in our dataset. With 449 daily observations, models with more than six breakpoints would generate very short segments, leading to unstable slope estimates and wide confidence intervals, thereby reducing interpretability. In addition, the AIC improved consistently up to the five-breakpoint model, while the six-breakpoint model showed no further improvement. Therefore, six breakpoints represented the empirical maximum applied in our analyses. Results of the segmented regression analyses are reported together with slope estimates, AIC values, breakpoint locations including 95% confidence intervals.

In Excel, the chosen model was again visualized and within the graph, a linear regression trendline was estimated for each segment (time between two breakpoints). For these regression lines, their respective slope and coefficient R² (explained variation) was stated. This information was used for further interpretation of data.

All costs were cumulated to calculate overall costs and divided by the number of applicants to calculate “cost per order”.

For the influencer marketing on social media in specific, an external marketing agency was responsible. This agency provided a report for each of the two flights including number of story or reel views, link clicks, website visits and resulting applications for participation. The costs of the influencer marketing measures were then set in relation to these indicators and resulted in specific costs per view, costs per click or finally, costs per order (application/first contact).

For analysing study applications over time there was no missing data. For 26 participants, information on the applied recruitment strategy was not available which is reported as “not specified” (Table 1). Consequently, no conclusions regarding the effectiveness of recruitment strategies can be drawn for these cases. However, these missing values represent only a minor proportion within each segment and are unlikely to materially affect the overall findings. Importantly, the estimation of breakpoints remained unaffected.

**Table 1:** Overview, analogue and digital recruiting measures and the respective recruiting outcomes, ranked by highest numbers of applications. #numbers refer to the listed recruiting measures in table 1. *For #1.1 and #1.2, the specific health magazine was not specified in the data

Analogue recruiting strategies	number of study applicants
#1: Short advertisements and articles in AOK BW health magazines (“young families” and “young women”)	total: 75 *#1.1 & #1.2: 54 #1.3: 11 #1.4: 10
#3: Flyers and personal addressment in AOK BW customer centres	6
#2: Flyers in gynaecological practices	5
#4: Flyers and personal addressment in AOK BW health centres	7
#5: Flyers in information centres for pregnant women and families in Baden-Wurttemberg	0
#6: Article in a local newspaper	0
#7: Personal approach to General Practitioners in Baden-Wurttemberg who partner with the AOK BW	0
total (analogue recruitment strategies)	93
Digital recruitment strategies	number of study applicants
#13: Influencer marketing on social media (Instagram)	total: 50 #13.1: 44 #13.2: 6
#10: Advertisement on the landing page of the AOK (www.AOK.de)	37
#15: Advertisement in two AOK Apps	19
#14: Social media campaign on AOK BW social media canals (Instagram and Facebook)	25
#11: Placement of native advertisements on different websites	2
#9: Articles and advertisement in different newsletters of the AOK BW	1
#12: Targeted mailing addressing women and men insured by the AOK BW aged 20–59	2
#8: Advertisement on information screens within the AOK BW customer centres	0
total (digital recruiting strategies)	136
not specified	26
Overall	255

## Results

**Fig. 1:**
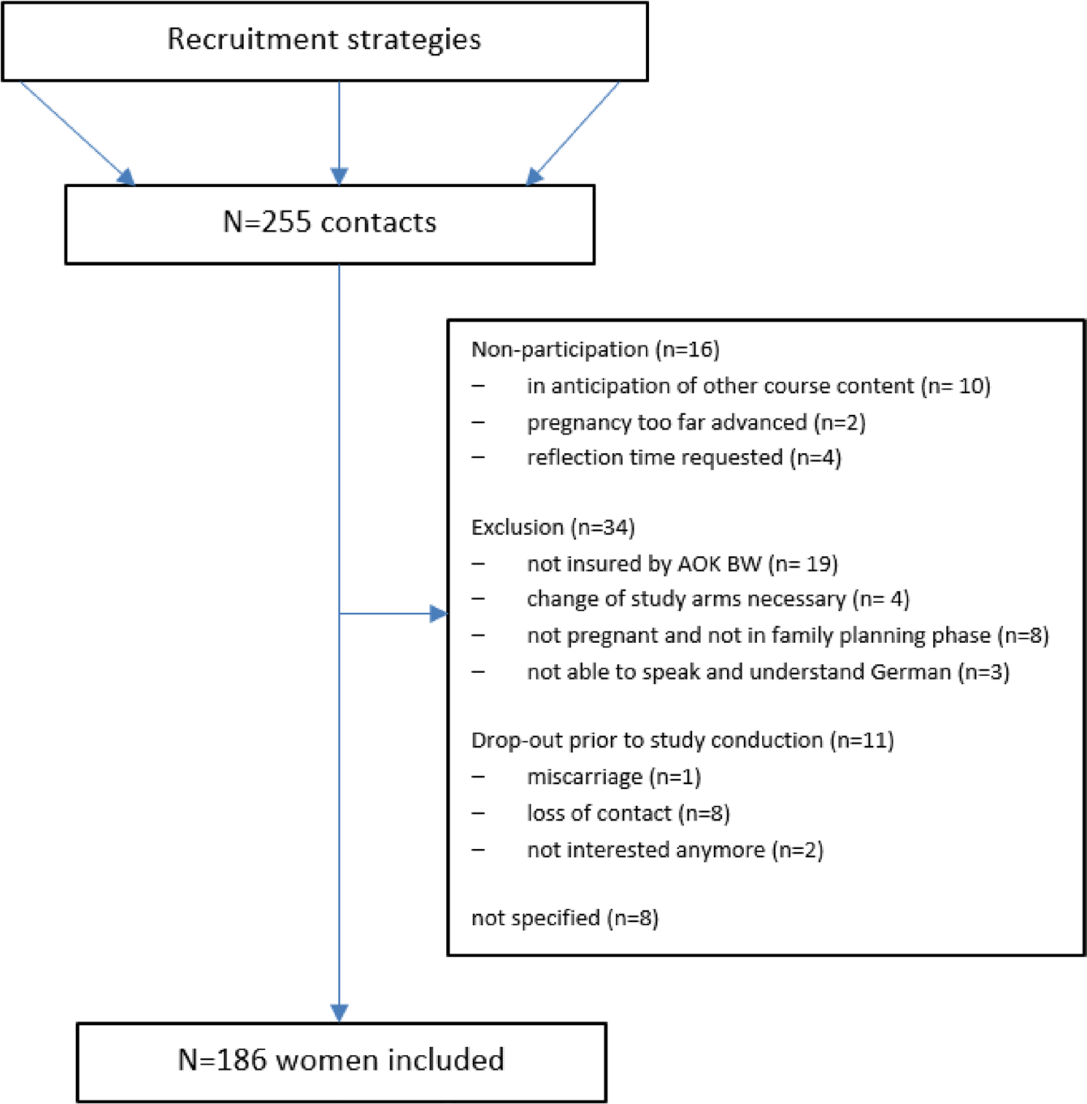
Flowchart of participant enrolment and reasons of exclusion

In total, *N* = 255 women contacted the study team and asked for study enrolment. Of these, 186 were enrolled in the study and randomised. Hence, the original target of 638 participants was only achieved by 29,1%, despite an extension of the intervention phase by nine months. Reasons for non-participation, exclusion or drop-out prior to conduction of further study procedures were: not being insured by AOK BW (exclusion criterion); not being in the family planning phase (exclusion criterion); dissatisfaction with the assigned study arm; learning that the consultation did not meet the expectations after receiving information about study components and procedures; miscarriage; or the participants cut off contact without naming any reasons (Fig. 1).

**Fig. 2:**
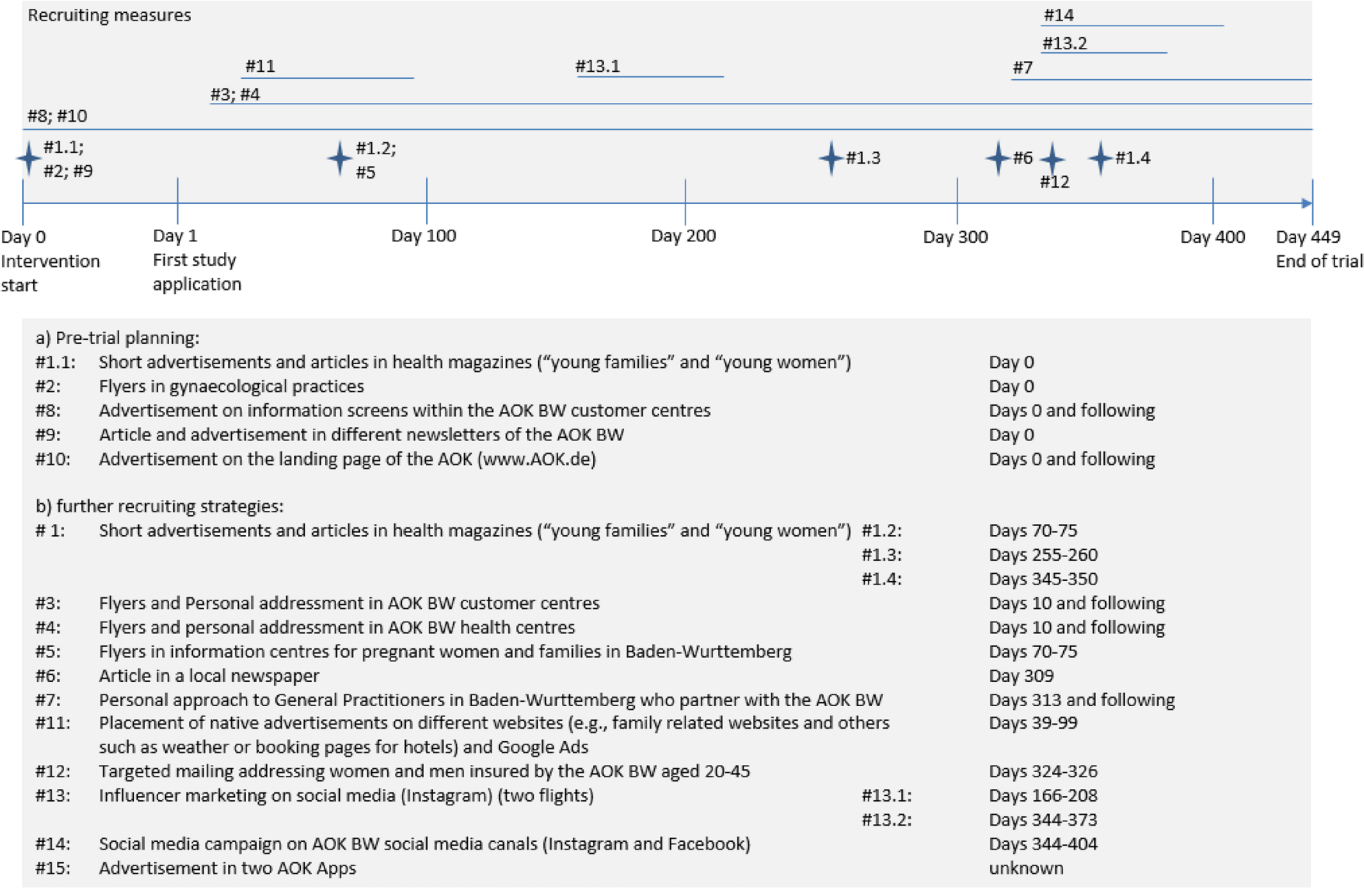
List of recruitment strategies within the AOK-Family + study and when they were applied

Figure 2 illustrates the timeline of all analogue and digital recruitment measures applied during the 449-day enrolment period, with the first participant enrolled on day 1 (24 April 2023) and the last on day 449 (15 July 2024). Recruitment activities clustered at distinct time points, reflecting the staggered implementation of strategies over the course of the trial.

Day 0 stands for the time period in advance to the intervention start (pre-trial planning) and the time period between the official intervention start (first of April 2023) and the first enrolment (24.04.2024). Day 1 marks the day of the first enrolment on 24.04.2023. The total intervention time was 449 days -measured from Day 1 (first day of study enrolment) and the last day of trial on 15.07.2024 which equals the last day of enrolment.

For data analysis of participant inflow, all 255 study applications were included in data analysis. Table 1 gives a detailed insight into the application inflow and recruiting outcomes.

The recruitment measures that resulted in the most (35,5%) study participants were the printed health magazines, which the health insurance company distributes to all insured persons by mail. Ranked second, the influencer marketing flights generated 23,6% of the study participants. Through personal approach by General Practitioners or the local newspaper article, no study participants were recruited. 1,6% of the participants reported they applied for study participation based on information flyers in gynaecological practices.

### Analysis of application inflow

Davies‘ test for significance of breakpoints resulted in a highly significant change in the slope of the participant inflow (*p*-value < 2.2e^− 16^). Results of the segmented linear regression analysis for different models with up to 6 breakpoints showed varying breakpoints for the 1- and 2- breakpoint models followed by consistent breakpoints at days 151, 209 and 323 for the other breakpoint models (Table 2). For the 5- and 6-breakpoint models, day 35 and 45/47 were consistent as well. Standard errors for the estimated breakpoints were lowest in the 2-, 3- and 5-breakpoint models. All six models showed a significant change of slope with their respective breakpoints via M-fluctuation-test. Together with this, the small standard errors of the specific breakpoints imply significant breakpoints in the 2-, 3-, 4-, 5-, and 6-breakpoint models.

**Table 2:** Statistic models with different numbers of breakpoints to analyse change in slope of application inflow. AIC = Akaike information criterion

Model	estimated breakpoints (Days)	Std. error of each breakpoint	Model parsimony (AIC)	Confidence interval for final model
0-breakpoint model	-	-	3.766,728	-
1-breakpoint model	373	21,66	3.759,528	-
2-breakpoint model	160	1,32	2.963,352	-
	196	1,31		
3-breakpoint model	157	0,66	2.351,115	-
	207	0,62		
	323	1,94		
4-breakpoint model	67	4,135	2.281,152	-
	151	0,65		
	209	0,60		
	323	1,79		
5-breakpoint model	35	1,61	2.222,445	[32,33; 38,64]
	47	1,64		[43,64; 50,07]
	151	0,59		[149,77; 152;06]
	209	0,56		[207,78; 209, 99]
	323	1,68		[319, 52; 326,09]
6-breakpoint model	35	1,63	2.225,154	-
	45	2,02		
	65	14,18		
	151	0,61		
	209	0,56		
	323	1,68		

**Fig. 3:**
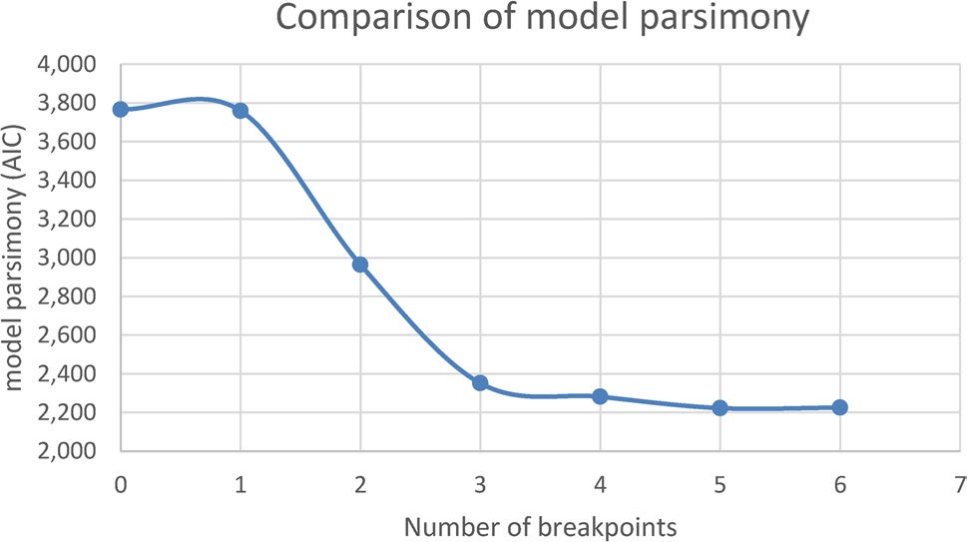
Comparison of model fit using AIC for models with 0,1,2,3,4,5 and 6 breakpoints

To identify the most plausible model with good fit and parsimony, models with up to 6 breakpoints were examined. We chose the five-breakpoint-model, mainly on the basis of the overall lowest AIC (2.222,445). Confidence intervals are shown for each breakpoint of the final 5-breakpoint model in Table 3. Figure 3 shows the highest decrease of AIC for the three-breakpoint-model with little but significant improvements for the 4- and 5-breakpoint models. The 6-breakpoint model showed an increase of AIC and no further improvement of model fit.

**Table 3:** Overview total costs of all recruitment strategies

Recruitment strategies	Costs
Analogue strategies	
1. Short advertisements and articles in health magazines (“young families”, print run of 454.450 and “young women”, print run of 406.085)	not specified
2. Flyers in gynaecological practices (30.000 printed flyers including mailing costs for 1.920 letters with 8 flyers each) additional Print/production/project management costs for 30.000 flyers	5.856,00 € 1788,45€
3. Flyers and Personal addressment in AOK BW customer centres	included in 2.
4. Flyers and personal addressment in AOK BW health centres	included in 2.
5. Flyers in information centres for pregnant women and families in Baden-Wurttemberg	included in 2.
6. Article in a local newspaper	not specified
7. Personal approach to General Practitioners in Baden-Wurttemberg who partner with the AOK BW	no costs
Digital measures	
8. Advertisement on information screens within the AOK BW customer centres	no costs
9. Article and advertisement in different newsletters of the AOK BW	not specified
10. Advertisement on the landing page of the AOK (www.AOK.de)	not specified
11. Placement of native advertisements on different websites (e.g., family related websites and others such as weather or booking pages for hotels) including agency costs Google Ads	21.204,56€ 15.001,00€
12. Targeted mailing addressing women and men insured by the AOK BW aged 20–45	not specified
13. Influencer marketing on social media (Instagram) flight #1 flight #2, including paid ads on Instagram and Facebook	34.272,00€ 20.706,00€
14. Social media campaign on AOK BW social media canals (Instagram and Facebook)	included in #13.
15. Advertisement in two AOK Apps	not specified
other costs: Further cooperate design costs for the AOK-Family + project	505,75€
total costs	99.333,76€

**Fig. 4:**
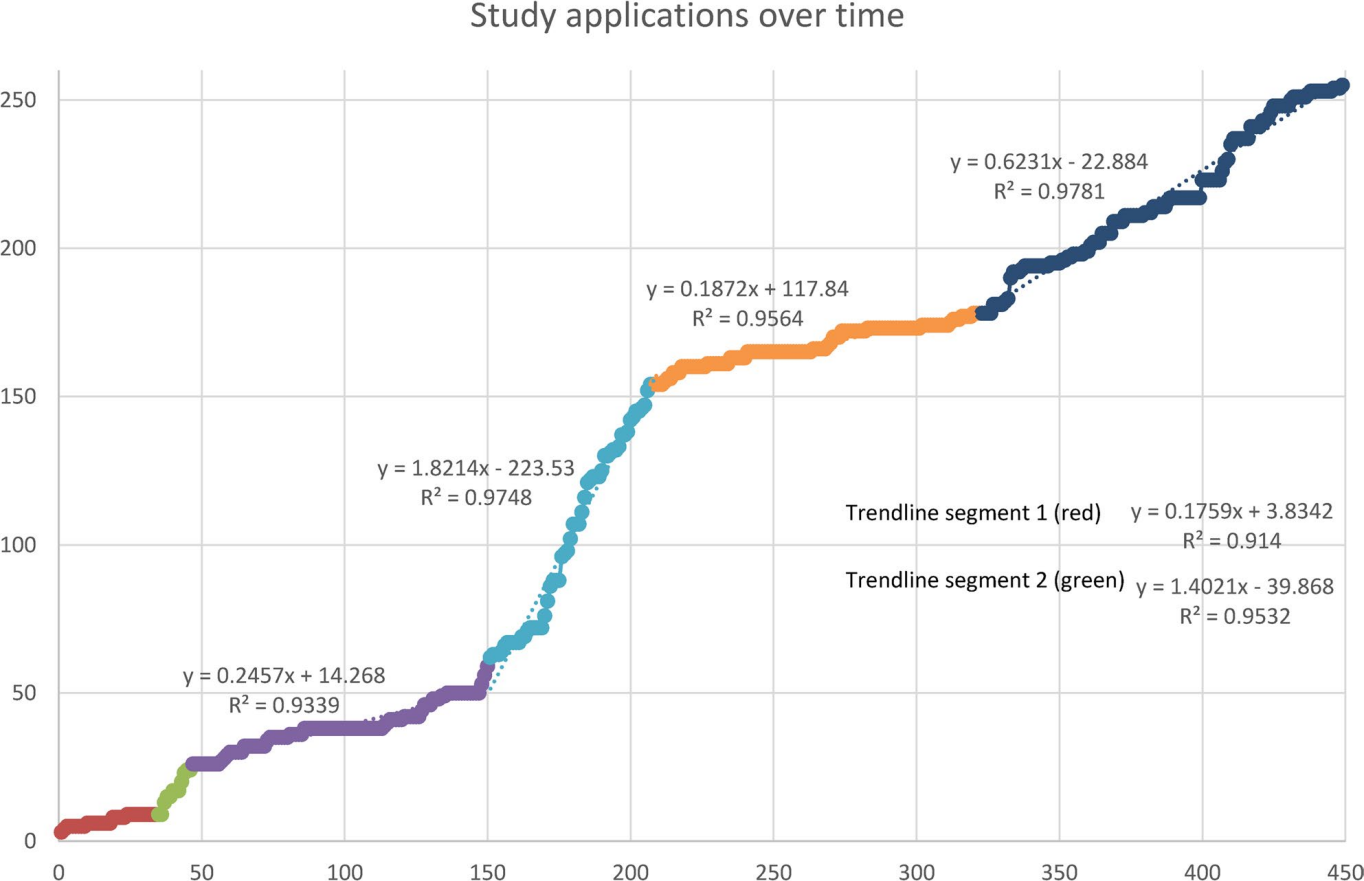
Application inflow (*n* = 255 women) over time (*n* = 449 days). Colours highlight the six segments parted by five breakpoints at days 35, 47, 151, 209 and 323. Each segment is added a linear regression trendline. This trendline is described using slope formula (“y=(…)”) and coefficient of determination (*R*²)

For further identification of the best model fit, application inflow is visualized in Fig. 3. In Fig. 4, the segmented regression results are displayed, with each segment distinguished by color. For ease of interpretation, the description of recruitment dynamics in the text explicitly refers to these colors (e.g., the red segment representing the initial recruitment phase,). The slopes and breakpoints reported correspond directly to the estimates shown in Fig. 4.

Here, five breakpoints are highlighted. Between the first three segments, a change of slope from 0,17 in the first segment to 1,4 in the second segment is observed implying a steep increase of study applications.

#### Segment 1 (Day 1 - Day 34; red)

Application inflow started slowly with a low elevation and slope of 0.18 (about one new participant every five to six days).

#### Segment 2 (Day 35 - Day 46; green)

The first breakpoint (day 35) showed a significant increase of the applications with a change of slopes from 0.18 in segment 1 to 1.4 in segment 2, equating to about one new participant every day. However, breakpoint 2 does not directly collide with the start or end of any of the recruiting strategies. Native ads were started on day 39 but were mentioned by only 2 applicants in total. When looking at the data in more detail, out of all 15 applications in this second segment, all but one applicant (93%) reported the health magazine as basis for their decision to call for study participation. Furthermore, only one reporting of the health magazine as recruitment strategies was observed before the first breakpoint on day 24. Even though the health magazine was described to be sent out earlier, it can be assumed that the health magazine actually was received later than day 1, caused the first change of slope and led to a steep increase of study applications.

#### Segment 3 (Day 47 – Day 150, purple)

Again, no recruitment strategy ends or starts around the time of the third segment starting with the second breakpoint. The first health magazine resulted in a significant increase in study applicants that lasted about 12 days and the applications then continued slowly, while no new recruitment strategy was conducted. Applications slowed to a slope of 0.24, corresponding to roughly one new participant every four days, largely explained by residual magazine exposure.

#### Segment 4 (Day 151 – Day 208; light blue)

 A significant change of slope is observed at breakpoint 3 (day 151). The third breakpoint indicated the strongest rise in recruitment, with the slope increasing to 1.8, equivalent to nearly two new participants per day. The fourth segment collides with the first influencer marketing flight which started day 166 and ended on day 208. Within this segment, 95 women applied for study enrolment. Out of these 95, 48 (51%) stated they gained interest from the first influencer marketing flight, 16 (17%) were assigned to the health magazines, 5 (5%) saw the ad on the AOK homepage and another 5 (5%) applicants reported the AOK App as basis for decision making. The remaining 21 either did not specify or reported isolated other measures. Combining these results, it can be observed that the first influencer flight was associated with the second peak of applications, but the breakpoint (start of increase) is before the social influencer campaign had started.

#### Segment 5 (Day 209 – Day 322; orange)

The slope significantly decreases within this segment from 1,8 in segment 4 to 0,19 in segment 5. Breakpoint 4 collides with the end of the first influencer marketing flight ending on day 208. Inflow dropped to a slope of 0.19, meaning about one new participant every five to six days, reflecting the immediate loss of recruitment momentum once the campaign ended.

#### Segment 6 (Day 323 – Day 449; dark blue)

The fifth and last breakpoint (day 323) is terminated shortly before a few measures: The second flight of influencer marketing (starting day 344), the social media campaign on Instagram and Facebook (starting day 344) as well as the fourth health magazine (mailed around day 345). Furthermore, the fifth breakpoint is shortly after the targeted mailing. However, this measure was only mentioned by one study participant in total. This last segment showed the last slight but significant increase in applications from previously 0,19 in the fifth segment to 0,62 in the sixth, corresponding to about one new participant every one to two days. As around this time many different recruiting strategies were performed, no specific recruitment strategy could be identified that might have caused this increase. Within this segment, 77 women applied for study participation. The recruitment strategies resulting in the most applicants were the health magazine (30%), the AOK website (21%), ads on Instagram or Facebook (16%) and the AOK App (12%).

This description of the recruiting process shows that the printed health magazines and the influencer marketing campaign on social media correlated with significant increases of study applications. The health magazines were mentioned by many applicants, also a longer period of time after its publication. Even though the social media posts were published over a greater period of time, the positioning of the breakpoints (moments at which the rate of recruitment increases) did not suggest a direct effect and recruitment decreased rapidly after the end of this respective period.

### Direct costs of recruitment strategies

On top of the recruiting outcomes, direct costs of the recruitment strategies are listed in Table 3. Indirect costs, for example, the costs of the health magazines or newsletters, were not considered. For adding an article or advertisement, there are no extra costs for internal projects besides design costs and working hours for text production which are part of the project.

All in all, a total spending of 99.333,76€ was documented for all analogue and digital measures for recruitment (Table 3). For 255 phone calls, this means a total cost per study application of 389,54€. When calculating the costs per order (cost per actual study participation) these costs increase to 534,05€ per participant.

Highest costs were caused by the influencer marketing flights and the native ads and Google ads. Within this first influencer flight on Instagram, three female influencers were included. Influencer 1 published a sequence of three Instagram Stories (a set of three consecutive images or short video clips), followed by a single reminder Story and an additional short video post (“reel”), which cost 7.497,00€ altogether. Influencer 2 also published a sequence of three Instagram Stories,), followed by a single reminder story and an additional short reel with the costs of 1.785,00€. Influencer 3 published one Instagram story and a reel for a total cost of 5.533,50€. Cost differences were due to the number of followers. Influencer 1 had 164.000 followers, influencer 2 20.600 and influencer 3 had 89.00 at that time. The influencer costs can be specified further with regards to the respective outcomes: All stories and reels resulted in a total of 789.919 views, 6.182 link-clicks, 2.382 website visits and 53 contacts. This means a cost per view of 0,024€, cost per website visit of 8,04€ and finally, the cost per order (first contact/application) of 390,71€. Within the second social media flight, the previous stories and reels were played out again and sponsored (paid ads) on Instagram and Facebook, which means that the content was published as ads for women in the targeted age group within their news feed. In total, 17.400,00€ were paid for these ads on Instagram (11.600,00€) and Facebook (5.800,00€). Together with agency costs and other additional costs, the second flight resulted in a total spending of 20.706,00€. As recruiting outcomes, a total of 534.492 story and reel views on Instagram, as well as 267.262 views on Facebook, a total of 25.461 link-clicks (cost per click of 0,68€, with reference to the net costs, without agency and additional spending) and 1.616 website visits were detected.

Compared to around 390€ per application via influencer marketing, the ads and articles in the AOK BW health magazines resulted in 75 study applications with no extra costs for the project.

## Discussion

This article gives detailed insights into the costs and efforts used for recruiting pregnant women and women planning to pregnant of childbearing age for an interventional study. Summarizing the main results, the targeted sample size was not reached despite substantial investment in recruitment measures. The first influencer marketing flight on Instagram is mentioned by a substantial number of participants, but the increase of recruitment rates seemed to start before start of the campaign and the recruitment rate decreased immediately with the ending of the campaign. It can be assumed that the printed health magazine also reached many thousands of women resulting in the highest number of applications over all measures and most likely to have caused the first steep increase of study applications. Digital measures via Instagram and Facebook initially attracted and engaged a high number of women, which may be a desirable effect by itself. However, only a few called for study participation. Analogue measures, and the health magazine in particular, were documented to be much cheaper and resulted in timely steadier study applications. Online ads and social media campaigns needed many resources and numbers of study applications declined rapidly after ending the efforts.

In general, it is seen as crucial to analyse needs of the target group to tailor recruitment measures individually [8, 33]. In this specific target group, challenges in committing spare time to participate in a trial might have been a barrier to participation [34]. Time-related constraints are perceived as a barrier for many pregnant women who are managing work, childcare, and household duties simultaneously. Recent research has shown that lack of time and competing responsibilities are among the most cited modifiable barriers to participation in physical activity interventions during pregnancy [35–37]. Especially for time-intensive offers requiring proactive engagement and direct phone contact—as in this study—this burden might disproportionately hinder participation. Evidence shows, trusted influential persons, such as healthcare providers, friends or partners might have a beneficial influence on study participation [7] and on a healthy behaviour during pregnancy [38]. Studies confirm that pregnant women often align their behaviors, including decisions about alcohol consumption, smoking, or healthy eating, with perceived family expectations and partner involvement [39, 40]. Within the process evaluation including consultants of the AOK-Family + project, participants believed in and suggested the engagement of care providers, specifically gynaecologists and midwifes, who could have acted as gatekeepers to address pregnant women in early pregnancy or even in preconception period [41]. This could imply a new care path for pregnant women and women in family planning phase through which the care provider suggests a consultation as soon as a pregnancy is diagnosed or family planning is discussed. Supporting this, health literacy levels in pregnant women is described as heterogenic and especially women with limited health literacy seem to rely on health care providers as a source of information [42]. Within the AOK-Family + study, involvement of gynaecologists was limited. Study flyers were sent to more than 1.900 gynaecological practices in south-western Germany via mail. However, it remains unclear, how many physicians actually displayed these flyers in their practice or approached women in person. As this measure only resulted in five applicants, it can be assumed that only few gynaecologists displayed the flyers or chose a personal approach to inform women of the target group about the consultation. For 2025, the close involvement of gynaecologists is planned within a following trial to promote implementation of the consultation. Whether this method will achieve a higher enrolment rate and enrolment of women in early pregnancy and preconception period should be assessed in a future study. As influencer marketing resulted in a significant increase of study applications, compared to only half of the application numbers via unpersonal social media advertisements, followed influencers might also have had a positive influence on the decision to participate in this study. However, privacy concerns can reduce trust in information received via social media in pregnant and postpartum women [43]. This is in line with findings suggesting that many women of reproductive age primarily use social media for personal rather than health-related purposes and perceive targeted health content with skepticism. Concerns about data privacy, distrust toward advertising, and discomfort with receiving sensitive health messaging via platforms like Facebook or Instagram may all contribute to resistance or disengagement [43–45].

Especially for pregnant women, health status is crucial within the first months of pregnancy [46] and quality of life seems to decrease from the fourth month onwards [47], which supports the importance of an early consultation to reduce LRRFs during pregnancy. For LRRF reduction, preconception period should be promoted as well, suggesting a higher focus on women in family planning phase [48]. On top of this, personal benefits and access to new innovative interventions might as well have a positive influence on the decision to participate [7], which was included in the central messages of advertisements in this trial. Advertisements on websites, in Apps or in magazines were the recruiting strategies, the most study applicants based their decision to participate on.

Other digital measures were ads on websites which accompanied the whole recruiting process over time, whereas analogue measures can only be published or sent out punctually. Interestingly, through social media content of both flights, a total of 31.643 link-clicks and 3.998 website visits could be achieved. However, this resulted in only 74 applications implying a loss of 98%. Specific reasons for this loss of potential participants could not be identified through social media analysis. One of the factors that might have had an influence on this outcome was the change of media due to a mandatory phone call instead of an online contact form. Interested women needed to visit the insurance company’s website and call the phone number, that was displayed, which could have created a barrier. Although the digital campaigns achieved considerable reach and a high number of clicks, only a small fraction translated into actual study applications. This suggests that the limited conversion was not primarily due to a “digital divide,” but rather to procedural and contextual barriers. First, the recruitment process required women to actively switch media and initiate a telephone call instead of completing a simple online form, which may have discouraged participation. Second, digital targeting was necessarily broad, and it is likely that many clicks came from women outside the specific eligibility criteria, resulting in high attrition between initial interest and actual enrolment. This could also contribute to the disparity between the wide digital reach and the relatively low conversion to actual participation observed in this study. This change of media can also be seen as a positive aspect for data protection, as several ethical issues of social media recruiting are discussed such as that there are no specific guidelines or regulations when planning social media recruitment [49–51]. Data protection issues include analysing deep insights of personal data of interested persons who clicked on a link or visited a study website. Within the AOK-Family + project, the social media analysis was done by an external agency and only aggregated data was presented to the study team. Furthermore, study applicants had to call the study team proactively which was independent of their website click and their application was not transparent to other social media users. Hence, these particular issues of data protection are not seen in this project. However, a proper ethical planning is recommended before the conduction of social media recruitment [52] which was not part of the project and should be considered in future studies.

One other factor of that might have contributed to the social media recruitment not being as effective as described in other studies might have been the very specific target group of the trial. Moreover, digital engagement does not automatically translate to action. Research shows that the intention-behavior gap is especially pronounced in health interventions, particularly if proactive steps like phone calls are required. While many women expressed initial interest in participation through online clicks or responses to digital campaigns, the conversion into actual enrollment was markedly lower. This discrepancy reflects the well-described *intention–behavior gap*, where intentions do not consistently translate into concrete actions. The INTACT-RS framework [53] provides a valuable lens for understanding this process by differentiating between intentional (motivational) and actional (volitional) components of behavior change, highlighting that supportive conditions and enabling factors are often required to bridge the gap between intention and actual participation.

The effort required to change platforms and actively initiate contact might have discouraged some participants, especially if they were uncertain about the benefits, time investment, or legitimacy of the study [54–57]. It can be assumed that women in childbearing age could have been reached but many did not meet these specific inclusion criteria such as the restricted geographical area and specific insurance requirements. Moreno et al. [58] aimed for a young target group and described similar experiences with the enrolment rate of potential study participants. They observed higher participation-rates through personal contact than social media despite a higher number of adolescents was reached through social media than via the in-person methods. Within the literature reviews of Darko et al. [12] and Sanchez et al. [13] the majority of included studies described social media recruiting to be cost-effective and adequate for reaching their recruitment target. Most of the studies who specified on social media recruitment included various measures such as paid-ads, native ads, and the inclusion of one or more social media platform, which were also used in this project but were not as cost-effective. Within the literature review of Darko et al. [12], half of the included studies who used social media for recruiting in health studies aimed to recruit the general population. But also, 38% of the studies aimed for hard-to-reach populations, including persons with alcohol and tobacco abuse or low health literacy [12], which is partly consistent with the target group of the AOK-Family + study. However, this was mostly reported for observational studies and especially geographically limited studies described in-person-recruiting to be more efficient and cost-effective than via social media [12, 58], which is in line with our findings and study setting. For now, it remains unclear to what extent the intervention reached high-risk women most in need of prevention, illustrating the prevention dilemma. In this study, cost per participation was rather high with more than 480€ per enrolment. Moreno et al. [58] for example described a cost per enrolment of 40 $ and preferred in-person methods for recruiting as a result. Consistent with this, Waltman et al. [59] aimed to recruit postmenopausal women and also found the in-person method to be the most effective. The highest number of enrolled participants was achieved by letters from care providers followed by postcard mailings. Within the review of Sanchez et al. [13], a median cost per study participant of 19,47$ is described and social media recruitment for mental health research is seen as more cost-effective compared to regular recruitment measures. Hence, reported experiences with social media recruitment in comparable research vary and within this study, costs were rather high with low recruitment outcomes.

For a better recruitment outcome, it is recommended to use a mixed-methods recruitment strategy including several social media platforms for digital content in combination with adequate analogue measures to recruit study participants [60]. Despite this is reflected by the reported recruitment strategy, the targeted number of participants was not reached by far which needs to be analysed further. One limitation was the absence of an initial budget specifically allocated to recruitment, particularly for social media content. A professionally planned and continuously implemented campaign might have yielded higher participation rates. Moreover, our recruitment process required women to switch media and initiate contact by telephone, which likely constituted an additional barrier. Future trials could reduce this friction by offering a direct online registration form embedded within digital advertisements or social media campaigns. Embedding referral links that capture the exact origin of each click would further allow precise tracking of recruitment sources. Together, these refinements could enhance both the efficiency of digital recruitment and the accuracy of process evaluation.

### Limitations

This study has several limitations. First, the reporting of recruitment sources was based on participant self-report at the time of initial contact. As women may have been exposed to multiple recruitment measures simultaneously, their attribution to a single measure could be subject to recall bias or misclassification. Consequently, the relative contribution of individual strategies might be over- or underestimated. Second, cost calculations did not include indirect expenses such as staff time for text production and design, which may affect cost-efficiency estimates. Third, segmented regression in this study is an explorative method to identify unknown breakpoints rather than testing a priori chosen ones. Breakpoints were identified directly from the data, and given uncertainties in the timing of recruitment measures, possible delays in participant response, and missing information on recruitment pathways, causal interpretations must be made with caution. Finally, the findings are derived from one health insurance setting in southwestern Germany and may not be fully generalizable to other populations or healthcare systems.

## Conclusions

As a conclusion, for recruiting pregnant women and women in childbearing age for a lifestyle intervention study, influencer marketing and articles in printed health magazines increased application numbers significantly. Analogue measures had lower costs with higher application numbers than social media recruitment. Despite a broad variety of digital and analogue recruitment strategies, the targeted number of participants could not be achieved. Social support from partners, family or healthcare providers may play a critical role in the decision to participate, underscoring the relevance of interpersonal and social dynamics during pregnancy. Despite high digital reach, actual conversion rates from social media were low. Engaging gynaecologists and midwifes as gatekeepers in an implemented care path could be one way to address the target group adequately and increase the use of a LRRF consultation in early pregnancy or preconception period. For future research and practice, combining broad digital outreach with personalized and trust-based communication—ideally via healthcare professionals such as gynecologists and midwives—may enhance acceptance and uptake. The involvement of gynecologists and midwives as key stakeholders is essential to reach women earlier, reduce participation barriers, and promote the use of consultations for reducing LRRFs in both the preconception period and early pregnancy.

## Data Availability

The datasets generated and analysed during the current study are not publicly available due to reasons of data safety but are available from the corresponding author on reasonable request.

## Abbreviations

LRRF: Lifestyle-related risk factors
AOK BW: AOK Baden-Wurttemberg (insurance company)
SES: Socioeconomical Status

## References

[1] Campbell MK, Snowdon C, Francis D, Elbourne D, McDonald AM, Knight R, et al. Recruitment to randomised trials: strategies for trial enrollment and participation study. Health Technol Assess. 2007;11(48):iiiix–105. The STEPS study. 10.3310/hta11480 17999843

[2] Carlisle B, Kimmelman J, Ramsay T, MacKinnon N. Unsuccessful trial accrual and human subjects protections: an empirical analysis of recently closed trials. Clin Trials. 2015;12(1):77–83. 25475878 10.1177/1740774514558307 PMC4516407

[3] Huang RC, Silva D, Beilin L, Neppe C, Mackie KE, Roffey E, et al. Feasibility of conducting an early pregnancy diet and lifestyle e-health intervention: the pregnancy lifestyle activity nutrition (PLAN) project. J Dev Orig Health Dis. 2020;11(1):58–70. 31391133 10.1017/S2040174419000400

[4] Di Carlo C, Iannotti G, Sparice S, Chiacchio MP, Greco E, Tommaselli GA, et al. The role of a personalized dietary intervention in managing gestational weight gain: a prospective, controlled study in a low-risk antenatal population. Arch Gynecol Obstet. 2014;289(4):765–70. 24129610 10.1007/s00404-013-3054-y

[5] Ronnberg AK, Ostlund I, Fadl H, Gottvall T, Nilsson K. Intervention during pregnancy to reduce excessive gestational weight gain—a randomised controlled trial. BJOG. 2015;122(4):537–44. 25367823 10.1111/1471-0528.13131

[6] Garnæs KK, Mørkved S, Salvesen KÅ, Salvesen Ø, Moholdt T. Exercise training during pregnancy reduces circulating insulin levels in overweight/obese women postpartum: secondary analysis of a randomised controlled trial (the ETIP trial). BMC Pregnancy Childbirth. 2018;18(1):18. 29310617 10.1186/s12884-017-1653-5 PMC5759335

[7] Houghton C, Dowling M, Meskell P, Hunter A, Gardner H, Conway A et al. Factors that impact on recruitment to randomised trials in health care: a qualitative evidence synthesis. Cochrane Database Syst Reviews. 2020(10). 10.1002/14651858.MR000045.pub2 PMC8078544 33026107

[8] Treweek S, Pitkethly M, Cook J, Fraser C, Mitchell E, Sullivan F et al. Strategies to improve recruitment to randomised trials. Cochrane Database Syst Reviews. 2018(2). 10.1002/14651858.MR000013.pub6 PMC7078793 29468635

[9] Fletcher B, Gheorghe A, Moore D, Wilson S, Damery S. Improving the recruitment activity of clinicians in randomised controlled trials: a systematic review. BMJ Open. 2012;2(1):e000496. 22228729 10.1136/bmjopen-2011-000496 PMC3253423

[10] Topolovec-Vranic J, Natarajan K. The use of social media in recruitment for medical research studies: a scoping review. J Med Internet Res. 2016;18(11):e286. 27821383 10.2196/jmir.5698 PMC5118584

[11] Whitaker C, Stevelink S, Fear N. The use of Facebook in recruiting participants for health research purposes: a systematic review. J Med Internet Res. 2017;19(8):e290. 28851679 10.2196/jmir.7071 PMC5594255

[12] Darko EM, Kleib M, Olson J. Social media use for research participant recruitment: integrative literature review. J Med Internet Res. 2022;24(8):e38015. 35925655 10.2196/38015 PMC9389385

[13] Sanchez C, Grzenda A, Varias A, Widge AS, Carpenter LL, McDonald WM, et al. Social media recruitment for mental health research: a systematic review. Compr Psychiatry. 2020;103:152197. 32992073 10.1016/j.comppsych.2020.152197 PMC7704547

[14] Parks AM, Duffecy J, McCabe JE, Blankstein Breman R, Milgrom J, Hirshler Y, et al. Lessons learned recruiting and retaining pregnant and postpartum individuals in digital trials: viewpoint. JMIR Pediatr Parent. 2022;5(2):e35320. 35107422 10.2196/35320 PMC9037306

[15] Marufu TC, Ahankari A, Coleman T, Lewis S. Maternal smoking and the risk of still birth: systematic review and meta-analysis. BMC Public Health. 2015;15:239. 25885887 10.1186/s12889-015-1552-5 PMC4372174

[16] Zhang S, Wang L, Yang T, Chen L, Zhao L, Wang T, et al. Parental alcohol consumption and the risk of congenital heart diseases in offspring: an updated systematic review and meta-analysis. Eur J Prev Cardiol. 2020;27(4):410–21. 31578093 10.1177/2047487319874530

[17] Navarro P, Mehegan J, Murrin CM, Kelleher CC, Phillips CM. Associations between a maternal healthy lifestyle score and adverse offspring birth outcomes and childhood obesity in the lifeways Cross-Generation cohort study. Int J Obes (Lond). 2020;44(11):2213–24. 32829383 10.1038/s41366-020-00652-x

[18] Saur AM, Dos Santos MA. Risk factors associated with stress symptoms during pregnancy and postpartum: integrative literature review. Women Health. 2021;61(7):651–67. 34311677 10.1080/03630242.2021.1954132

[19] Organization WH. Strengthening legal and regulatory frameworks for maternal and perinatal death surveillance and response. 2024. 66 p.

[20] Melo P, Coomarasamy A, Coomarasamy A. Pregnancy and childbirth risks: clinical and legal perspectives. 2023. pp. 237 – 56.

[21] Teede HJ, Bailey C, Moran LJ, Bahri Khomami M, Enticott J, Ranasinha S, et al. Association of antenatal diet and physical activity-based interventions with gestational weight gain and pregnancy outcomes: a systematic review and meta-analysis. JAMA Intern Med. 2022;182(2):106–14. 34928300 10.1001/jamainternmed.2021.6373 PMC8689430

[22] Russo LM, Nobles C, Ertel KA, Chasan-Taber L, Whitcomb BW. Physical activity interventions in pregnancy and risk of gestational diabetes mellitus: a systematic review and meta-analysis. Obstet Gynecol. 2015;125(3):576–82. 25730218 10.1097/AOG.0000000000000691

[23] Bonello K, Figoni H, Blanchard E, Vignier N, Avenin G, Melchior M, et al. Prevalence of smoking during pregnancy and associated social inequalities in developed countries over the 1995–2020 period: a systematic review. Paediatr Perinat Epidemiol. 2023;37(6):555–65. 37427978 10.1111/ppe.12989

[24] Camier A, Kadawathagedara M, Lioret S, Bois C, Cheminat M, Dufourg MN et al. Social inequalities in prenatal folic acid supplementation: results from the ELFE cohort. Nutrients. 2019;11(5). 10.3390/nu11051108 PMC6566921 31109064

[25] Sun J, Piernicka M, Worska A, Szumilewicz A. A socio-ecological model of factors influencing physical activity in pregnant women: a systematic review. Front Public Health. 2023;11:1232625. 38054068 10.3389/fpubh.2023.1232625 PMC10694207

[26] Yu Y, Feng C, Bédard B, Fraser W, Dubois L. Diet quality during pregnancy and its association with social factors: 3D cohort study (Design, Develop, Discover). Matern Child Nutr. 2022;18(4):e13403. 35821643 10.1111/mcn.13403 PMC9480933

[27] Hobel J, Müters S. Sozioökonomischer Status und Gesundheit: Datenlage, Befunde und Entwicklungen in Deutschland. 2024.

[28] Kaba-Schönstein L, Kilian H. Gesundheitsförderung und soziale Benachteiligung / Gesundheitsförderung und gesundheitliche Chancengleichheit. In: (BZgA) BfgA, editor. Leitbegriffe der Gesundheitsförderung und Prävention Glossar zu Konzepten, Strategien und Methoden. 2023.

[29] Bonevski B, Randell M, Paul C, Chapman K, Twyman L, Bryant J, et al. Reaching the hard-to-reach: a systematic review of strategies for improving health and medical research with socially disadvantaged groups. BMC Med Res Methodol. 2014;14:42. 24669751 10.1186/1471-2288-14-42 PMC3974746

[30] Krämer M, Wohlhüter L, Hermeling L, Koetsenruijter J, Kamradt M, Wensing M, et al. A counselling intervention for individual strategies to prevent complications and strengthen resources during pregnancy in gynaecological care (AOK-Family +): study protocol for a cluster-randomised controlled trial. Trials. 2024;25(1):393. 38890726 10.1186/s13063-024-08215-5 PMC11186123

[31] R. A language and environment for statistical computing. R Foundation for Statistical Computing; 2023.

[32] Muggeo VM. Estimating regression models with unknown break-points. Stat Med. 2003;22(19):3055–71. 12973787 10.1002/sim.1545

[33] Marcano Belisario JS, Bruggeling MN, Gunn LH, Brusamento S, Car J. Interventions for recruiting smokers into cessation programmes. Cochrane Database Syst Rev. 2012(12). 10.1002/14651858.CD009187.pub2 PMC6485998 23235672

[34] Rokicki S, Gobburu A, Weidner M, Azam N, Jansen M, Rivera-Núñez Z, et al. Barriers and strategies for recruitment of pregnant women in contemporary longitudinal birth cohort studies. BMC Med Res Methodol. 2025;25(1):117. 40295914 10.1186/s12874-025-02570-w PMC12036123

[35] Connelly M, Brown H, van der Pligt P, Teychenne M. Modifiable barriers to leisure-time physical activity during pregnancy: a qualitative study investigating first time mother’s views and experiences. BMC Pregnancy Childbirth. 2015;15:100. 25896111 10.1186/s12884-015-0529-9 PMC4409747

[36] Coll CVN, Domingues MR, Gonçalves H, Bertoldi AD. Perceived barriers to leisure-time physical activity during pregnancy: a literature review of quantitative and qualitative evidence. J Sci Med Sport. 2017;20(1):17–25. 27372276 10.1016/j.jsams.2016.06.007

[37] Alrzeghi N, Elbsheni F. Perceived barriers to physical activity during pregnancy. J Womens Health. 2022;10(10).

[38] van Lonkhuijzen RM, Rustenhoven H, de Vries JHM, Wagemakers A. The role of the partner in the support of a pregnant woman’s healthy diet: an explorative qualitative study. BMC Pregnancy Childbirth. 2023;23(1):760. 37898778 10.1186/s12884-023-06072-9 PMC10612286

[39] Nierop A, Bratsikas A, Zimmermann R, Ehlert U. Are stress-induced cortisol changes during pregnancy associated with postpartum depressive symptoms? Psychosom Med. 2006;68(6):931–7. 17132840 10.1097/01.psy.0000244385.93141.3b

[40] Rini CK, Dunkel-Schetter C, Wadhwa PD, Sandman CA. Psychological adaptation and birth outcomes: the role of personal resources, stress, and sociocultural context in pregnancy. Health Psychol. 1999;18(4):333–45. 10431934 10.1037//0278-6133.18.4.333

[41] Litke N, Ullrich C, Wohlhüter L, Wensing M, Bombana M. Lifestyle-Related Counselling during Pregnancy in a Health Insurance Setting: A Qualitative Process Evaluation Study in Germany (submitted manuscript). BMC Pregnancy and Childbirth; 2025.

[42] Nawabi F, Krebs F, Vennedey V, Shukri A, Lorenz L, Stock S. Health literacy in pregnant women: A systematic review. Int J Environ Res Public Health. 2021;18(7). 10.3390/ijerph18073847 PMC8038834 33917631

[43] Hao H, Lee YW, Sharko M, Li Q, Zhang Y. Privacy concerns versus personalized health content-pregnant individuals’ willingness to share personal health information on social media: survey study. JMIR Form Res. 2025;9:e60862. 39930996 10.2196/60862 PMC11833185

[44] Frennesson NF, McQuire C, Aijaz Khan S, Barnett J, Zuccolo L. Evaluating messaging on prenatal health behaviors using social media data: systematic review. J Med Internet Res. 2023;25:e44912. 38117557 10.2196/44912 PMC10765287

[45] John JN, Gorman S, Scales D, Gorman J. Online misleading information about women’s reproductive health: a narrative review. J Gen Intern Med. 2025;40(5):1123–31. 39511120 10.1007/s11606-024-09118-6 PMC11968640

[46] Poon LC, McIntyre HD, Hyett JA, da Fonseca EB, Hod M. The first-trimester of pregnancy – a window of opportunity for prediction and prevention of pregnancy complications and future life. Diabetes Res Clin Pract. 2018;145:20–30. 29852233 10.1016/j.diabres.2018.05.002

[47] Morin M, Claris O, Dussart C, Frelat A, de Place A, Molinier L, et al. Health-related quality of life during pregnancy: a repeated measures study of changes from the first trimester to birth. Acta Obstet Gynecol Scand. 2019;98(10):1282–91. 30985917 10.1111/aogs.13624

[48] Stephenson J, Heslehurst N, Hall J, Schoenaker DAJM, Hutchinson J, Cade JE, et al. Before the beginning: nutrition and lifestyle in the preconception period and its importance for future health. Lancet. 2018;391(10132):1830–41. 29673873 10.1016/S0140-6736(18)30311-8 PMC6075697

[49] Ferrigno BN, Sade RM. Ethics of recruiting research subjects through social media. Am J Bioeth. 2019;19(6):73–5. 31135321 10.1080/15265161.2019.1602192 PMC7244227

[50] Gelinas L, Robin P, Sabune W, Glenn CI, Fernandez LH, Bierer BE. Using social media as a research recruitment tool: ethical issues and recommendations. Am J Bioeth. 2017;17(3):3–14. 28207365 10.1080/15265161.2016.1276644 PMC5324729

[51] Bender JL, Cyr AB, Arbuckle L, Ferris LE. Ethics and privacy implications of using the internet and social media to recruit participants for health research: a privacy-by-Design framework for online recruitment. J Med Internet Res. 2017;19(4):e104. 28385682 10.2196/jmir.7029 PMC5399223

[52] Zimmermann BM, Willem T, Bredthauer CJ, Buyx A. Ethical issues in social media recruitment for clinical studies: ethical analysis and framework. J Med Internet Res. 2022;24(5):e31231. 35503247 10.2196/31231 PMC9115665

[53] Lander J, Heiberger A, Von Sommoggy J, Schulz AA, Dresch C, Altawil H, et al. Intentional and actional components of engaged participation in public health research studies: qualitative synthesis of a recruitment and retention process into the theory-informed INTACT-RS framework. BMC Med Res Methodol. 2023;23(1):17. 36647023 10.1186/s12874-023-01838-3 PMC9841138

[54] Rhodes RE, de Bruijn GJ. How big is the physical activity intention-behaviour gap? A meta-analysis using the action control framework. Br J Health Psychol. 2013;18(2):296–309. 23480428 10.1111/bjhp.12032

[55] Sheeran P. Intention—Behavior relations: a conceptual and empirical review. Eur Rev Social Psychol. 2002;12(1):1–36.

[56] van Gemert-Pijnen JE, Nijland N, van Limburg M, Ossebaard HC, Kelders SM, Eysenbach G, et al. A holistic framework to improve the uptake and impact of eHealth technologies. J Med Internet Res. 2011;13(4):e111. 22155738 10.2196/jmir.1672 PMC3278097

[57] Webb TL, Sheeran P. Does changing behavioral intentions engender behavior change? A meta-analysis of the experimental evidence. Psychol Bull. 2006;132(2):249–68. 16536643 10.1037/0033-2909.132.2.249

[58] Moreno MA, Waite A, Pumper M, Colburn T, Holm M, Mendoza J. Recruiting adolescent research participants: in-person compared to social media approaches. Cyberpsychol Behav Soc Netw. 2017;20(1):64–7. 27976951 10.1089/cyber.2016.0319

[59] Waltman NL, Smith KM, Kupzyk KA, Lappe JM, Mack LR, Bilek LD. Approaches to recruitment of postmenopausal women for a community-based study. Nurs Res. 2019;68(4):307–16. 30829836 10.1097/NNR.0000000000000356 PMC6602805

[60] Goldman N, Willem T, Buyx A, Zimmermann BM. Practical benefits, challenges, and recommendations on social media recruitment: multi-stakeholder interview study. J Med Internet Res. 2023;25:e44587. 37213177 10.2196/44587 PMC10242465

